# Obesity management: at the forefront against disease stigma and therapeutic inertia

**DOI:** 10.1007/s40519-021-01217-1

**Published:** 2021-05-29

**Authors:** Luca Busetto, Paolo Sbraccia, Roberto Vettor

**Affiliations:** 1 Department of Medicine, University of Padova, Padua, Italy; 2 Department of Systems Medicine, University of Rome Tor Vergata, Rome, Italy; 3 Clinica Medica 3, Azienda Ospedale-Università di Padova, Via Giustiniani 2, 35128 Padua, Italy

**Keywords:** Obesity, Stigma, Chronic diseases, Therapeutic inertia

## Abstract

Obesity is a complex chronic relapsing disease, resulting from the interaction between multiple environmental, genetic and epigenetic causes, and supported by changes in the neuroendocrine mechanisms regulating energy balance and body weight. Adipose tissue dysfunction contributes to obesity-related complications. However, the prevalent narrative about the causes and mechanisms of obesity remains a much more simplistic one, based on the false assumption that individuals can fully control their body weight through appropriate behavioural choices. According to this narrative, obesity is simply reversible “persuading” the patient to follow healthier and more virtuous individual behaviours (moral judgement). This persistent narrative forms the deep root of the stigmatisation of people with obesity at the individual level and creates a clear discrepancy on how obesity prevention and cure are designed in comparison with the case of other non-communicable chronic diseases (clinical stigma). The promotion of systemic preventive measures against obesity is not supported at a political and social level by the persistence of a narrative of obesity as the simple consequence of individual failures and lack of willpower. The simplistic narrative of obesity as a self-imposed condition with an easy way-out (“eat less and move more”) creates a clear discrepancy on how obesity is managed by health care systems in comparison with other NCDs. The over-estimation of the efficacy of therapeutic intervention solely based on patients education and lifestyle modification is responsible of therapeutic inertia in health care professionals and in clinical guidelines, limiting or delaying the adoption of more effective therapeutic strategies, like anti-obesity medications and bariatric surgery. In conclusion, the persistence of a narrative describing obesity as a self-induced easily reversible condition has profound consequences on how obesity prevention and management are build, including the design and implementation of obesity management guidelines and a tendency to therapeutic inertia.

Level of evidence: No level of evidence.

## Obesity as a chronic disease and stigma against obesity

Nowadays, many international medical organizations and scientific societies consider obesity as a complex chronic relapsing disease, resulting from the interaction between multiple environmental, genetic and epigenetic causes, and supported by changes in the neuroendocrine mechanisms regulating energy balance and body weight [1]. Single-gene mutations or polygenic traits interact with environmental factors (dietary elements, sedentariness and lack of physical activity, sleep disturbances or deprivation, chronic stress, drugs or endocrine disruptors) altering the normal functioning of the neuroendocrine mechanisms regulating food intake, energy expenditure and the energy balance. By consequence, energy intake increases more than expenditure and fat accumulation ensues. The critical role of the hypothalamic circuits in causing obesity is proven by the fact that all the rare forms of severe obesity arising from single-gene mutations are related to alterations in genes involved in the normal functioning of the circuits, like leptin, leptin receptor, pro-opiomelanocortin (POMC), prohormone subtilisin/kexin 1 convertase (PCSK1) and melanocortin receptor (MC4-R) [2]. Moreover, the vast majority of genes whose subtle alterations have been linked to increased body mass index (BMI) or adiposity in genome wide association studies are mainly located into the central nervous system [3]. By consequence, patients with obesity are characterised by alterations in the mechanisms involved in the regulation of food intake, mainly satiety, and they involuntarily tend to eat more than they need when exposed to foods [4]. Therefore, patients with these subtle alterations are more susceptible to gain weight and accumulate fat when they became exposed to psychological stress or conditions favouring frequent eating or simply living in the food-promoting and physical activity-discouraging “obesogenic” environment in which all we lives in these times.

Moreover, when fat accumulated, the activation of numerous physiological mechanisms opposing weight loss and favouring weight regain counteracts any attempt to lose it [5]. Gut hormone secretion during weight loss is modified according to a usual pattern of compensatory changes: reduction in anorectic hormones secretion and increase in orexigenic hormones. These changes lead to both increased appetite and reward value of food leading to increased energy intake [5]. In addition, resting energy expenditure after weight loss is lower than expected according to body composition changes. This gap between observed and predicted energy expenditure following weight loss, named “metabolic adaptation”, could favour weight regain [5]. This complicated scenario, beyond patient motivation, makes weight regain a challenge in long-term management interventions in patients with obesity and configures obesity as a chronic relapsing disease [1].

As described above, the mechanisms leading to obesity development are primarily located at the central nervous system. However, adipose tissue participates in the regulation of body weight and its dysfunction is critical in the development of obesity complications. Adipose tissue is deeply involved in the regulation of energy balance and food intake, by modifying its response to different internal and external stimuli, such as energetic availability, physical activity and external temperature. In response to variation in energy storage, adipose tissue is able to modify its endocrine activity and anatomical characteristics, like cell size and number through hypertrophy and hyperplasia, and cell type through the *browning* process, converting white adipose tissue (WAT) into brown adipose tissue (BAT). In a condition of energy excess, when the increase in dimension and number of adipocytes reaches a critical, predefined threshold, WAT modulates an endocrine response to limit further energy intake exceeding the storing capacity through the release of circulating adipokines (leptin) acting on the central neural centers of appetite [6]. On the other hand, energy restriction promotes the reprogramming of cellular metabolism in adipose tissue, inducing mitochondrial biogenesis, and FFA oxidation instead of glucose oxidation. This phenomenon is also observed in WAT after cold exposure, which, through sympathetic stimulation, can activate thermogenic adipocytes (BAT or beige adipose tissue) to use stored fat for thermogenesis. When cold stimulation breaks off, thermogenic adipocytes can revert to a white phenotype. In summary, both WAT and BAT are main players of adaptive processes regulating energy balance. However, when this flexibility is altered, or in the presence of a condition of maladaptation to the metabolic and/or energetic stimuli, the regulatory mechanisms became defective with possible abnormal responses. Adipose tissue dysfunction, the so-called *adiposopathy*, is characterized by several abnormalities and first of all by morphological and functional changes. A large increase in adipocyte size and a decrease in the number and adipogenic potential of adipose stem cells characterize adipose tissue in both type 2 diabetes and prediabetes [7], limiting the progressive healthy expansion of adipose tissue through hyperplasia. This dysfunctional hypertrophic adipose tissue is not able anymore to store lipids efficiently, and this leads to ectopic lipid deposition in liver, skeletal muscle and heart, with negative effects on the function of these organs and a further deterioration in whole body metabolism [8]. Dysfunctional adipose tissue is also characterized by dysregulation of the mitochondrial biogenesis, infiltration from inflammatory cells and altered adipokines production pattern [9]. The production of adipokines contributes to the systemic pro-inflammatory state associated with obesity and has important adverse actions on the cardio-vascular system. In addition to their direct effects on pathophysiological processes in the cardio-vascular system, adipokines can affect cardio-vascular risk indirectly by modulating metabolism in liver, skeletal muscle and heart. These abnormalities express the loss of the integrity of the regulatory mechanisms of energy balance, predisposing to the development of obesity and obesity-related complications.

Notwithstanding these recent advances on the comprehension of the patho-physiologic mechanisms causing and maintaining obesity in the long-term, the prevalent narrative about the causes of obesity in the general audience, the media, the policy-makers, the health-care professionals and the patients with obesity themselves remains a much more simplistic one. According to this persistent narrative, individuals can fully control their body weight through appropriate behavioural choices, and therefore overweight and obesity appear as the direct consequence of inappropriate individual behaviours characterised by laziness, gluttony, and so on [10]. According to this narrative, obesity is simply reversible “persuading” the patient to follow healthier and more virtuous individual behaviours (moral judgement). This narrative is not supported by scientific evidence and points against modern evidence that, as stated above, delineates potent biological mechanisms as causes of weight gain and frequent weight rebound, classically attributed to the patient’s lack of willingness [10].

This persistent narrative forms the deep root of the stigmatisation of people with obesity in any social domain. If obesity is the consequence of inappropriate personal behaviours, patients with obesity are entirely responsible for their condition, and obesity is a sign (“stigma”) of lack of willpower and individual failure. Weight stigma has been documented in all social areas, including workplaces, schools, the family, and healthcare organisations [11, 12]. Overweight children are frequently mocked and bullied at school, and overweight or obese teenagers are very often isolated and are likely to be exposed to incidents of verbal, physical, or cyber discrimination [13]. Weight-related stigma is also common among people with obesity itself (“internalised stigma”). In the United States, about 40 to 50% of people with obesity show aspects of stigma internalisation and this is especially common amongst patients with high BMI levels who are trying to lose weight [14]. Mass media and social media are a pervasive source of weight-related stigma, especially as a result of the use of inappropriate images which portray people with obesity as lazy, greedy, dirty, sweaty, clumsy and even stupid [15]. It has been estimated that more than two-thirds of the images accompanying obesity reports in the U.S. media contain stigmatising content, and experimental studies have demonstrated that the viewing of these images increases the extent of the stigma [16]. Unfortunately, obesity stigma also exists among healthcare professionals, including family doctors, endocrinologists, cardiologists, nurses, dietitians, psychologists, medical students, and also professionals directly involved in the research or treatment of obesity [17, 18].

Weight-related stigmatisation exerts profound consequences at the individual level. For people living with obesity, exposure to weight-related stigmatisation and discrimination is a risk factor for mental disorders, which can be more severe than the obesity itself. Exposure to stigma is a risk factor for depression, high anxiety levels, low self-esteem, stress and substance abuse [19–22]. Stigma is also associated with an increased risk of dietary changes, such as binge eating disorders and the tendency to overeat in response to emotional factors [22]. Paradoxically, experimental studies have shown that exposure to weight-related stigma can lead to increased food intake [23]. Observational and interventional studies have also shown that the experience of weight-related stigmatisation is also associated with low levels of physical activity [24–26], the adoption of unhealthy dietary habits and a sedentary lifestyle [23]. The stigmatisation of weight and discrimination against people suffering from obesity, are associated with a further tendency to gain weight [26] and an increased risk of progressing from an overweight condition to a condition of obesity [28–30]. Finally, people with overweight and obesity who have experienced weight-related discrimination have higher levels of C-reactive protein [31] and cortisol [32], and a higher mortality rate [33] than people of the same weight who have not suffered discrimination.

Beside its deleterious effects on the mental and physical health of people with obesity, the stigma or “social disapproval” related to obesity, as a result of stereotyping and the use of inappropriate language and images, has led to an incorrect and negative portrayal of the disease itself. If body weight is under the voluntary control of the patient, the cure for obesity is simply to tell the patient that he needs to “eat less and exercise more”. Therefore, the implementation of most effective and more complex interventions become unnecessary. Recent international research, involving both individuals with obesity and the physicians treating them (ACTION-IO study), have shown a widespread overestimation of the effectiveness of interventions based solely on paternalistic advice and simple behavioural prescriptions on both sides [34, 35]. This was at the expense of other interventions (structured lifestyle modification programmes, cognitive behavioural therapy, drug treatment, surgical therapy) whose efficacy is supported by clear experimental evidences. Therefore, the push for a greater diffusion and availability of these interventions is considered useless. This simplistic and superficial view of the problem creates a clear discrepancy on how obesity prevention and cure are designed in comparison with the case of other non-communicable chronic diseases (NCDs). Individual behavioural choices are equally important for the genesis and maintenance of obesity as well as they are for any other NCD (type 2 diabetes, cardiovascular diseases, chronic lung obstructive pulmonary disease, et cetera) and several forms of cancer. However, only in the case of obesity, prevention and treatment have been based solely on individual self-care. The existence of this clinical stigma appears unjustifiable and indefensible from medical, ethical and social points of view [10, 36].

## Obesity stigma, prevention and research

The overestimation of the effectiveness of interventions based solely on paternalistic advice had profound consequences on the implementation of effective preventive measures to curb obesity epidemic so far. If body weight is under the voluntary control of people, prevention of weight gain and obesity should be based simply on the willingness of individuals to “eat less and exercise more”. Years and years of failures of preventive efforts relying mostly on individual counselling and education, and the continuing increase of obesity prevalence worldwide, should convince on the contrary. The curb of tobacco use and the incidence of smoking-related diseases has been obtained by coupling educational initiatives with more systemic approaches, like increasing the cost of cigarettes throughout taxation and regulating advertisements of tobacco products and smoking in public areas [37]. Of course, obesity is not equal to tobacco-related diseases, but several systemic approaches with proved efficacy for obesity prevention has been already identified [38]. According to World Health Organization, this should include implementation of evidence-based and population-based policies that make regular physical activity and healthier dietary choices available, affordable and easily accessible to everyone, particularly to the poorest individuals. Examples of such a policy are taxation on sweetened beverages, nutritional labelling of foods that can educate consumers toward the consumption of more healthier dietary patterns, restricting marketing of unhealthy foods, especially those aimed at children and teenagers, and ensuring the availability of healthy food choices and supporting regular physical activity practice in the workplace [38]. The implementation of these systemic preventive measures is not without political costs and involves several stakeholders with important economic interests. The willingness and power to promote and enforce these systemic measures is surely not supported at a political and social level by the persistence of a narrative of obesity as the simple consequence of individual failures and lack of willpower.

If body weight is under the voluntary control of the patient, and treating obesity merely involves telling the patient to “eat less and exercise more”, it makes no sense to invest resources into the research and implementation of new effective therapies for the treatment of obesity. In the United States, investments by the National Institutes of Health for cancer and AIDS research are 5–10 times higher than the expected investment in obesity, despite the fact that obesity is the most common chronic disease among American citizens [10]. There is a close correlation between weight stigma and the willingness to invest in obesity research. The ASK study, conducted in the United Kingdom, the United States, Australia, and New Zealand, involved 5623 subjects: 1567 healthcare professionals and 4,056 subjects from the general population. The study clearly showed that people who had more stigmatizing and discriminatory attitudes against people with obesity were also those who were less likely to increase spending on obesity research [38]. The same study evidenced a strong correlation between the perception that obesity can be cured simply by following a healthy lifestyle and a higher stigma score [39]. In this context, research to clarify the etiological mechanisms of obesity is therefore not clearly perceived as a priority. Furthermore, funding may only be diverted towards projects that are seen as effective (implementation of behavior and lifestyle interventions), reducing support for the research of new methods of prevention and treatment, and the implementation of therapies (anti-obesity medications or bariatric surgery) already available that are effective and safe according to scientific evidences [10].

## Therapeutic inertia

As stated before, the simplistic narrative of obesity as a self-imposed condition with an easy way-out (“eat less and move more”) creates a clear discrepancy on how obesity is managed by health care systems in comparison with other NCDs. Indeed, many healthcare systems, both public and private, do not offer patients with obesity the same level of care given to other chronic diseases (such as cancer, diabetes, cardiovascular and rheumatic diseases) [10]. In Italy, patients with obesity do not benefit from forms of exemption of health-care costs as provided by our regulation for other chronic diseases. Moreover, patients with obesity have limited access to therapeutic education and intensive lifestyle modification programmes in the national health system, and cognitive behavioural therapy programmes are rarely offered. Furthermore, none of the drugs specifically indicated for obesity therapy are available free of charge, and access to bariatric surgery, with treatment pathways involving a multidisciplinary follow-up, is very difficult to obtain in certain parts of the country. Finally, the availability and accessibility of outpatients or inpatients multidimensional rehabilitation programmes for patients with the most severe and disabling forms of obesity is very also limited. These numerous forms of obesity stigma in the healthcare sector, which lead to patients with obesity having fewer safeguards and fewer opportunities for treatment than patients with other chronic diseases, can be classified under the term clinical stigma.

We also believe that the definition of obesity as a self-induced easily reversible condition and the over-estimation of the efficacy of therapeutic intervention solely based on patients education and lifestyle modification is responsible of therapeutic inertia in health care professionals. In most of obesity management guidelines published so far, the possibility to use more effective therapeutic strategies, like anti-obesity medications and bariatric surgery, was limited to patients with higher BMI values or with slightly lower BMI only in the presence of established obesity-related comorbidities or complications. In current European guidelines for obesity management in adults [40], bariatric surgery can be considered in patients with BMI ≥ 40 kg/m^2^, but in patients with BMI 35.0–39.9 kg/m^2^ only in the presence of comorbidities. In analogy, anti-obesity medications can be prescribed in patients with BMI ≥ 30 kg/m^2^, but in patients with BMI 25.0–29.9 kg/m^2^ only in the presence of comorbidities. Why should we wait the occurrence of complications before intensifying therapeutic strategy, instead of intensifying therapeutic strategy in patients with high risk of complications in order to prevent their occurrence? The typical case here is that of patients with prediabetes, in which the risk of developing type 2 diabetes is highly increased. We have evidence for both anti-obesity medications [41] and bariatric surgery [42], that a more intensive body weight control leads to a significant reduction of the risk of developing type 2 diabetes in this high risk population, with the prevention of around 4 cases of type 2 diabetes in every 5 patients. Why should we wait the occurrence of type 2 diabetes before intensifying therapeutic strategy, instead of intensifying therapeutic strategy in patients with prediabetes in order to prevent the metabolic disease?

The over-estimation of the efficacy of therapeutic intervention solely based on education and lifestyle modification in comparison with the proven efficacy of more intensive therapeutic strategies impacts also in the development and implementation of the guidelines for obesity management published so far. In the European guidelines for obesity management in adults [40], lifestyle modifications, anti-obesity medications and bariatric surgery are presented as possible initial levels of intervention to discuss with the patient. In other words, guidelines specify, according to BMI levels, fat distribution and the presence of comorbidities, in which patients more intensive therapeutic strategies (anti-obesity medications and bariatric surgery) *can* be used, but they do not specify in which patients these interventions *should* be used or are recommended. This is at stark difference with how the guidelines for other NCDs are developed and they work. In the guidelines for the management of arterial hypertension, lifestyle advice can be used alone only in patents with high normal or grade 1 blood pressure levels and not at high risk for cardio-vascular diseases, but immediate drug treatment is recommended since the beginning in patients with higher blood pressure levels and/or high cardiovascular risk [43]. In analogy, in the management of dyslipidaemias, intervention strategy is recommended according to the starting LDL-cholesterol levels and cardio-vascular risk. Lifestyle intervention is recommended alone as the starting therapeutic strategy in patients combining low LDL-cholesterol levels and low cardiovascular risk, with lipid-lowering drugs added only if lipids levels remain uncontrolled. However, immediate drug intervention coupled with lifestyle modifications is recommended since the beginning in patients presenting with high LDL-cholesterol levels and/or high or very high cardiovascular risk [44]. We believe that obesity management guidelines should be more adherent to these models and less liberal in the choice of therapeutic interventions. This is a claim for adapting the intensiveness of the therapy to the clinical characteristics of the patients, leaving less space for the patients or physicians preferences. The proposal of a lifestyle modification programme alone in a patient with severe and/or complicated obesity, long obesity history and repeated weight cycling is a form of therapeutic inertia that it is no longer clinically acceptable.

Therapeutic inertia, has been here defined as a failure to start or intensify therapy when indicated by clinical guidelines. Diagnostic inertia is the failure to recognize a patient as affected by a disease, like the case of a patient without known hypertension and high blood pressure labelled “normal” by medical staff [45]. This example recalls the case of obesity labelled as “healthy” when the disease is not accompanied by manifested and codified disorders of glucose and lipid metabolism and/or cardiovascular complications, despite several large observations demonstrating that individuals with “healthy” obesity are at a higher risk for cardiovascular events, type 2 diabetes and all-cause mortality than people with normal weight [46]. This is again the consequence of a lack of understanding obesity as a disease and the continuity of its pathological processes. A further proof of this is provided by the behavior of regulatory agencies allowing a divergent reimbursement system for identical pharmacological active compounds used in different ways and with different brand names in obesity or in diabetes.

Questions remain whether the lack of starting or delay in putting forward an intensified alternative therapeutic strategy for patients not reaching an adequate BMI target represents true inappropriate care or whether it is an acceptable decision to prevent the risks of overtreatment in particular clinical cases and settings. The latter has been termed “appropriate inaction”—in opposition to “inappropriate inertia”—and is considered to be a condition favoring the lack to add a drug treatment or to shift to bariatric surgery, or in other words to provide a treatment intensification in clinical practice. In obesity, where clinical guidelines advocate a patient-centered approach, is it possible to consider an appropriate inaction? Are there complex patients (e.g., the elderly or individuals with associated severe life-threatening diseases) in which the obesity state could be a favorable clinical situation leading to avoid any weight loss program? This could be the case in some patients or in some group of patients, but it could not apply to the majority of people living with obesity in which even a moderate weight loss has proven medical, physical and mental benefits [40].

Within this broader definition of clinical, diagnostic and therapeutic inertia, the failure to advance treatment may have both short- and long-term consequences. Therapeutic inertia could be considered a missed opportunities to prevent complications at early stages (i.e. the progression from prediabetes to type 2 diabetes or/and from NASFLD to NASH) or reduce the risk of long term complications (i.e. heart failure or sleep apnea syndrome). Therapeutic inertia is a barrier to effective treatment and a common and widespread problem; it typically affects the care of most of people with obesity at every stage in the natural history of this disease. If we sum up delays from all treatment steps (awareness of the problem by the patient, and by the care provider, the decision to start with any treatment and the decision to intensify treatment and step up to drug or bariatric surgery) patients may spend many years with an unappropriated level of BMI before to start with an appropriate treatment. Failure to start or intensify treatment leads to avoidable delays, which in turn result in increased “bad” metabolic memory or excessive body weight legacy from excessively long periods of obesity, eventually increasing the risk of obesity-related complications. Much of the inertia in addressing obesity can be attributed to the prevailing and persistent framing of obesity as matter of personal responsibility. This prejudice manifests both as overt fat-shaming and as conscious or unconscious bias, including health care professionals and policy makers who should be providing and supporting care [47].

## Conclusion and actions for changing

**Fig. 1:**
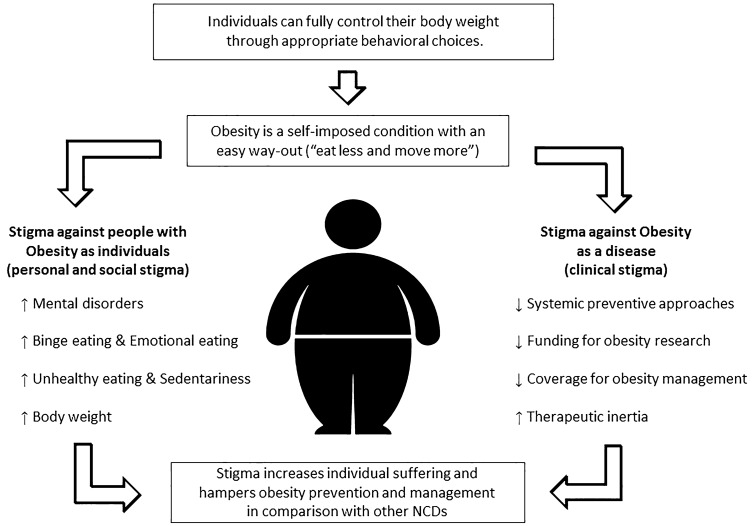
The cascade of stigma. According to a persistent simplistic narrative, individuals can fully control their body weight through appropriate behavioural choices, and therefore obesity appears as a self-imposed condition with an easy way-out (“eat less and move more”). This persistent narrative forms the deep root of the stigmatisation of people with obesity in any social domain (personal and social stigma) and causes individual suffering. Moreover, this narrative creates a clear discrepancy on how obesity is prevented and managed by health care systems as compared with was is done for other non-communicable chronic diseases (NCDs) (clinical stigma). The combination of personal, social and clinical stigma increases individual suffering and hampers obesity prevention and management

In conclusion, the persistence of a narrative describing obesity as a self-induced easily reversible condition has profound consequences on the burden of suffering of people with obesity and on how obesity prevention and management are build, including the design and implementation of obesity management guidelines and a tendency to therapeutic inertia (Fig. 1).

What actions should be devised in order to change this scenario? The following can be proposed and pursued:

1. Increasing awareness of obesity as a complex, chronic disease with strong biological bases at any levels, and in particular among healthcare professionals, both by including ad hoc sections into the training curriculum of medical and surgical students, and healthcare profession graduates, and by promoting continuing medical education events on this topic.
2. Interaction within scientific societies and professionals organisations, policymakers, patients and consumers organisations, and relevant stakeholders for the promotion of obesity preventive measures more based on a systemic approach and less limited to the change of individual behaviours.
3. The adoption of European and national regulatory initiatives to define obesity as a chronic disease characterised by high economic and social costs, both direct and indirect. In Italy, a Parliamentary Motion asking for this recognition was approved by a unanimous vote of the Italian Chamber of Deputies on November 11th, 2019. However, obesity is still not included in the list of chronic diseases for which the Italian National Health System has a mandate to care for and build specific plans for prevention and management.
4. Promoting the creation and implementation of multidisciplinary specialist structures for obesity management at the regional level, possibly organised in networks, able to provide patients with obesity with all the levels of treatment currently recommended in national and international guidelines for obesity management, including structured lifestyle modification programmes, psychological and behavioural therapy, drug therapy, and bariatric surgery.
5. The production and dissemination of obesity management guidelines more adherent to the concept of obesity as a chronic disease and including more stringent recommendations about the required levels of care according to the patients clinical characteristics and the therapeutic goals.
